# 

**Published:** 1904-03

**Authors:** 


*THE BRISTOL MEDICO-CHIRURGICAL SOCIETY.*

### CONTENTS.

PAGE

**Leprosy in Jamaica.** By W. D. NEISH, M.D. and T. J. TONKIN, L.R.C.P., L.R.C.S. Edin. ... 1

**Subungual Exostosis.** By W. ROGER WILLIAMS, F.R.C.S. ... 17

**Aneurysm of the Ascending Aorta.** By F. H. EDGEWORTH, M.B. Cantab., D.Sc. Lond. ... 23

**The Vagaries of Abdominal Tuberculosis: A Clinical Study.** By JAMES SWAIN, M.S., M.D. Lond., F.R.C.S. ... 31

**The Present Position of Radiotherapy in Therapeutics.** By W. KENNETH WILLS, M.B., B.C. Cantab. ... 39

**A Case of Tubal Gestation Producing Severe Hemorrhage without Rupture, Associated with the Presence of an Accessory Fallopian Tube.** By ERNEST W. HEY GROVES, M.D., B.Sc. Lond. ... 46

**Ethyl Chloride as a General Anæsthetic.** By C. HAMILTON WHITEFORD, M.R.C.S., L.R.C.P. ... 49

PAGE

**Note on the Subsequent History of a Case of Pylorectomy.** By A. W. PRICHARD ... 53

**Mortality and Sickness in the City of Bristol for the Year 1903.** By D. S. DAVIES, M.D. Lond. ... 55

**Progress of the Medical Sciences:**

**Medicine.** THEODORE FISHER, M.D. Lond., M.R.C.P. ... 58

**Surgery.** J. LACY FIRTH, M.S., M.D. Lond., F.R.C.S. ... 62

**Reviews of Books** ... 68

**Editorial Notes** ... 74

**Notes on Preparations for the Sick** ... 81

**The Library of the Bristol Medico-Chirurgical Society** ... 85

**Meetings of Societies** ... 87

**Local Medical Notes** ... 94

